# Changes in Seed Oil Profile and Morphological Characteristics in Sunflower Cultivars Under Salinity Stress and Nanoparticle Treatments

**DOI:** 10.1002/fsn3.70968

**Published:** 2025-09-14

**Authors:** Shiva Shariatzadeh, Seyed Mehdi Talebi, Kimia Anjomani, Mansour Ghorbanpour

**Affiliations:** ^1^ Department of Biology, Science and Research Branch Islamic Azad University Tehran Iran; ^2^ Department of Research Landscape and Green Space Organization Shahinshahr Iran; ^3^ Department of Biology, Faculty of Science Arak University Arak Iran; ^4^ Department of Medicinal Plants, Faculty of Agriculture and Natural Resources Arak University Arak Iran

**Keywords:** fatty acid, *Helianthus annuus* morphometry, nanomaterials, phytochemistry

## Abstract

This study investigated the effects of zinc oxide and iron oxide nanoparticles and two salinity levels on the morphological characteristics and seed fatty acid profiles of two sunflower hybrids (G1601 and Shams). The research aimed to understand the impacts of these treatments/stresses potentially leading to strategies for improving crop resilience in water‐scarce environments, especially considering their potential roles as fertilizers and stress mitigators. This experiment utilized a randomized complete block design with a factorial arrangement (nanoparticle type, salinity levels, hybrid type, and age) of five treatments (50 and 150 mM salinity, 20 ppm ZnO and Fe_2_O_3_ nanoparticles, and control samples) with 20 replications. Morphological measurements for the root, leaf, stem, and seed dimensions were taken using a digital caliper. For both hybrids, seed priming with Fe_2_O_3_ nanoparticles resulted in the highest root dimensions, while 150 mM salinity stress led to the lowest values for these parameters. For both hybrids, the highest and smallest yielded seeds belonged to the control and 150 mM salinity‐stressed plants, respectively. In the G1601 hybrid, salinity stress (50 mM) produced the largest leaves, whereas Fe_2_O_3_ in second, third, and sixth week and ZnO nanoparticles in fourth and fifth week resulted in the smallest. In the Shams hybrid, the smallest leaves were recorded for the Fe_2_O_3_ nanoparticles treated in first to fourth week and the control plants in fifth to seventh week, while the biggest leaves belonged to the control in first to second week and ZnO nanoparticles treated samples in third, fifth, and seventh week. The Shams hybrid seed oil analysis showed linoleic acid as the dominant fatty acid (63.29%–72.98%), followed by oleic acid (15.33%–22.78%). Conversely, the G1601 hybrid seed oil was characterized by oleic acid as the primary fatty acid (46.54%–80.34%), with linoleic acid presented at a lower percentage (8.67%–40.29%). In the Shams hybrid, oleic acid amount significantly increased in all the treated/stressed samples, except for those that were subjected to 50 mM salinity stress. Conversely, linoleic acid amounts decreased in these salinity‐stressed samples. Meanwhile, in the G1601 hybrid, linoleic acid content increased significantly, while oleic acid content decreased in the treated/stressed samples. Additionally, the total percentages of saturated and unsaturated fatty acid amounts changed under these treatments/stresses. The responses of sunflower plants to nanoparticles and salinity stresses were not uniform; they varied based on several factors, including the plant's genetic structure (hybrid type), age, and the specific type of nanoparticle or salinity treatment/stress applied. Developing more tolerant sunflower hybrids requires understanding how different treatments affect fatty acid biosynthesis involved genes and how hybrids respond differently. Therefore, investigations into the effect of various treatments/stresses and expression of involved genes are recommended for future works.

## Graphical abstract

This study investigated the effects of zinc oxide and iron oxide nanoparticles, along with NaCl salinity stress, on the morphology and seed oil fatty acid profile of two sunflower cultivars, G0601 and Shams. The findings indicate that nanoparticle treatments and salinity stress significantly impacted both morphological characteristics and fatty acid composition, with notable differences between these cultivars. The variability in cultivar responses to nanoparticles/salinity stress is influenced by several factors, including the plant's genetic structure, age, and environmental conditions.

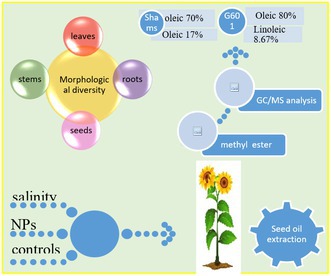

## 1 Introduction

The common sunflower, *Helianthus annuus* L., is an economically important annual plant in the Asteraceae family. Native to North America, it was later introduced and cultivated globally for its oil, seeds, and other uses (Talebi et al. 2024). The seed of sunflower yields about 30%–50% oil, contains 20%–30% proteins, and variable amounts of other compounds such as tocopherols. Additionally, a great part of this oil is composed of unsaturated fatty acids, which rise up to 90% in some hybrids (Rauf 2019). Alberio et al. (2017) indicated that the improvement and modification of oils are the important breeding goals in sunflower plants. Breeding programs aim to develop sunflower hybrids that improve oil quality and quantity, enhance resistance to stress, and diversify oil production for various purposes. These goals include increasing oil yield and reducing saturated fatty acids percentages (OECD 2015), strengthening resistance against diseases and drought (Talebi et al. 2024), and creating a range of oils for different applications (Shah et al. 2023). For example, oil with high linoleic acid content is more suitable for use in salad dressings (Chernova et al. 2021), and oils with a high level of saturated fatty acids exhibit more stability during cooking and deep frying (Romano et al. 2021).

The combination of climate change, population growth, and industrial development is leading to a global freshwater shortage, making the use of saline water for agriculture increasingly necessary. This shift is driven by the rising demand for water in agriculture, coupled with reduced availability of freshwater resources (El‐Bially et al. 2022).

Salinity stress negatively impacts plant growth by causing cell water loss, potentially leading to plant death. This stress can manifest in several ways, including decreased stem height, leaf number and size, biomass, and seed production (Han et al. 2022). Salinity stress can reduce oil yield, alter the oleic/linoleic acids ratio, and increase the proportion of unsaturated fatty acids (Céccoli et al. 2022). Additionally, the percentages of palmitic and stearic acids, both saturated fatty acids, can be increased (Noreen and Ashraf 2010). Salinity stress in sunflowers decreases water uptake, negatively impacts photosynthesis, reduces chlorophyll and carotenoid contents, impairs gaseous exchange, and stunted growth, which ultimately decreases the amount of oil production (Ashraf and Siddiqi 2024; Younis and Mansour 2024).

Salinity stress significantly impacts plant lipid metabolism, carbon assimilation, and induces oxidative stress, which leads to variation in seed oil profiles. These effects are linked to ion toxicity, disruptions in seed development, and imbalances in cellular redox homeostasis (Taher et al. 2018; Jahanshahi et al. 2024). The impact of environmental factors on plants can differ greatly depending on the specific genetic structure (genotype or hybrid) of the plant, as highlighted by research conducted by Pan et al. (2021) and Céccoli et al. (2022).

Nanoparticles can significantly impact plant growth and development, with effects ranging from beneficial to detrimental, depending on some parameters, such as concentration and type of nanoparticles, species, and environmental condition (Feng, Jan, and Seiler 2022; Feng, Kreslavski, et al. 2022). At optimal levels, they can enhance photosynthesis (improve the efficiency of the Calvin‐Benson cycle, and activity of enzymes like Rubisco), nutrient uptake, and overall biomass by influencing various physiological and morphological processes. However, excessive nanoparticle concentrations can induce oxidative stress, reduce chlorophyll content, and impair growth and development (Talebi 2024).

Iron (Fe) is a crucial micronutrient with significant roles in plant biology, notably in chlorophyll biosynthesis, organelle development, and photosynthesis. It acts as a cofactor for numerous enzymes involved in these processes, making it vital for plant growth and health (Ning et al. 2023). Moreover, this element mediates the synthesis of RNA and certain enzymes (Sági‐Kazár et al. 2023). Iron oxide nanoparticles offer advantages over bulk iron or iron oxide due to their sizes and surface properties, leading to increased iron ion availability for plants. Their smaller size allows for a higher surface area, facilitating interactions with plant cells and enhanced nutrient uptake. Additionally, these nanoparticles can be engineered to form complex structures with other molecules, further optimizing iron delivery to plant organs (Poddar et al. 2020).

A prior study on sunflower seeds revealed that zinc oxide and iron oxide nanoparticles significantly influence both seed germination rate and the total polyphenol content in the resulting plants. These nanoparticles can either enhance or inhibit germination depending on the specific size and concentration. Additionally, the nanoparticles can affect the concentration of polyphenols, which are important phytochemicals, impacting the overall phytochemical profile (Hafizi and Nasr 2018; Al‐Sudani et al. 2024).

Several studies have been conducted on the effects of salinity stress (Han et al. 2022; Bakhoum et al. 2020) and nanoparticle treatment on seed germination, morphological and phytochemical characteristics, oil yield, and composition of sunflower (Al‐Sudani et al. 2024; Kornarzyński et al. 2020), while specific studies on the combined effects of salinity stress and nanoparticle treatment on morphological characteristics and seed oil composition of certain sunflower hybrids are limited.

The introduction of new sunflower hybrids is rapid, with many being registered annually, but there is a lack of in‐depth research on their performance in stressful conditions like salinity (Stepasyuk et al. 2024). Specifically, Shams and G1601 hybrids, among others, have not been thoroughly studied for their morphological and oil composition responses to saline environments, or how they might benefit from nanoparticle treatments. This gap in knowledge highlights the need for research to understand how these new hybrids might adapt and potentially thrive in challenging conditions and increase their production under nanoparticle treatments.

This study aimed to investigate the effects of diverse levels of NaCl salinity stress, zinc oxide, and iron oxide nanoparticle treatments on two sunflower hybrids, Shams and G1601. The research will focus on how these treatments impact seed oil fatty acid profiles and plant morphology. Specifically, the study will examine variations in fatty acid profiles under different stress/treatments, the correlations between treatments/stress and fatty acid variations, the influences of these stresses/treatments on morphological characteristics, and assess differences in morphological and phytochemical responses between these hybrids under various conditions. The research was unique because it examined the specific sunflower hybrids Shams and G1601 and their combined response to NaCl salinity and ZnO/Fe_2_O_3_ nanoparticles. Moreover, no prior study has investigated these hybrids with these specific nanoparticle treatments and saline conditions, either in Iran or globally.

## 2 Material and Methods

### 2.1 Seed Cultivation, Nanoparticles, and Salinity Treatment

Seeds of sunflower hybrids were obtained from Seed and Plant Certification and Registration Institute (Agricultural Research, Education and Extension Organization, Ministry of Agriculture, Karaj‐Iran). Shams (Ha107F81‐112) was improved by crossing between CMS line AF81‐112 and restorer line RF81‐82 using the simultaneous selection method (Ghaffari et al. 2019).

Seeds were disinfected with a 2% Carbendazim solution for 5 min, followed by three washes with sterile water (Shen et al. 2014). For priming, 100 seeds of each hybrid were soaked in distilled water (control), 50 and 150 mM NaCl salinity levels, and 20 PPM of Fe_2_O_3_ and ZnO nanoparticles at 10°C for 7 days (Table 1). The nanoparticles concentrations were chosen according to previous investigation (Pirzada et al. 2022). The choice of 10°C for 7 days is a frequently used setting for priming sunflower seeds. This temperature range is often chosen because it is considered a relatively low, but not extremely stressful, temperature for sunflower seeds. The 7‐day duration allows sufficient time for the seed to imbibe the priming solution and initiate the physiological changes associated with germination (Pavitramata et al. 2023).

**TABLE 1:** The used factors and their levels for treatment of sunflower hybrids.

No.	Factors	Levels	Characteristics	CAS number and company
1	Salinity stress	50 mM	Molecular weight 58.44	7647‐14‐5, Sigma‐Aldrich
2	Salinity stress	150 mM	Molecular weight 58.44	7647‐14‐5, Sigma‐Aldrich
3	Fe2O3 nanoparticles	20 ppm	crystal structure 30 nm	1317‐61‐9, Sigma‐Aldrich
4	ZnO nanoparticles	20 ppm	crystal structure 75 nm	1314‐13‐2, Sigma‐Aldrich

The seeds were first placed between two moistened filter papers, then transferred to pots containing a 70/30 blend of Coco and Perlite. These pots were transferred to a greenhouse to grow under conditions on a 10 h light (25°C ± 3°C) and 14 h dark (20°C ± 3°C) cycle (He et al. 2024). One plant was kept per pot to create an appropriate density. After 4 weeks of cultivation, one‐half of Hoagland solution was utilized for plants feeding and irrigation. The study was designed to investigate the effects of two NaCl salinity levels (50 and 150 mM) and two nanoparticle treatments (Fe_2_O_3_ and ZnO, both at 20 Parts Per Million‐PPM) on the morphological characteristics and seed oils fatty acid profile. The experimental design involved a factorial approach with 20 replications per nanoparticle treatment and salinity conditions. The salinity treatments were performed by application of NaCl (Sigma‐Aldrich Company) solutions to the pots containing seedlings. To mitigate osmotic shock from salinity stress, plants were exposed to a gradual increase in salinity over three steps and simultaneously treated with nanoparticles through leaf spraying at sunset on a weekly basis for 7 weeks. In order to induce salinity stress, saline solutions (50 and 100 mM) were added to the soil a for each salinity stress level; gradually increasing the concentration was performed over time (20, 30, 50 mM, or higher) to simulate increasing salinity stress. It is a common method for inducing salinity stress in plants (Gupta et al. 2024).

Nanoparticles were sprayed onto plant leaves via foliar application using an aerosol delivery method. This technique, known as aerosol spraying, involves dispersing nanoparticles into a fine mist that is then applied to the plant's foliage. The nanoparticles are absorbed by the plant, potentially improving nutrient uptake, pest control, or other beneficial effects (Hong et al. 2021). Control plants received distilled water and were grown under identical conditions except for the salinity and nanoparticle treatments (Bami et al. 2021).

### 2.2 Morphological Examination

Measurements were taken on various plant parts at different times: rootlet length and diameter at the first week, and stem and seed dimensions at the seventh week. Leaf length and width were measured weekly using a digital caliper, with all measurements recorded in millimeters (mm). For each treatment/stress group, 20 individuals were measured for each morphological trait.

#### 2.2.1 Seed Oil Extraction

About 5 g (g) of mature and intact sunflower seeds from the control, nanoparticles, and salinity treated/stressed plants were processed to extract oils. The seeds were cleaned, ground, cooked, and dried. An automated extraction system (FOSS Soxtec 2055) was used, with n‐hexane (Sigma‐Aldrich) as the solvent (seed: solvent ratio of 1:10). After initial boiling at 70°C for 25 min (immersion phase), the samples underwent rinsing cycles for 30 min, followed by solvent (fresh n‐hexane) recovery for 15 min and final drying at 50°C for 10 min under vacuum. The extraction procedure was repeated three times to improve oil recovery (Kostic et al. 2013).

#### 2.2.2 Preparation of Fatty Acid Methyl Ester

The extracted oil samples were transferred into a three‐neck round‐bottom flask and preheated at 70°C for 30 min. Then the prepared potassium hydroxide (Sigma‐Aldrich), methanol (Sigma‐Aldrich) and n‐hexane solutions were added to the preheated oil samples, and the mixture was agitated at 500 rpm at 65°C for 30 s. We boiled the mixtures at 70°C for 2 min. Then, HCl (Sigma‐Aldrich) was added for neutralization. Following the transesterification of oils, the resulting mixtures were allowed to settle in a separating funnel for 24 h. This gravity settling process separated the glycerol (a byproduct) from the fatty acid methyl esters (FAME) phase. The collected ester‐containing phase was washed with hot distilled water (around 50°C) to remove any remaining impurities, such as traces of catalyst, methanol, soaps, or glycerol. Subsequently, the mixture of methanol and water was evaporated using a rotary evaporator at 70°C, effectively removing both components and leaving behind the purified ester phase (Salimon et al. 2014; Zamba and Reshad 2022).

#### 2.2.3 GC and GC–MS Analyses

Gas chromatography (GC: 6890N Agilent, USA) and GC–MS (HP6890GC/5973MS Agilent Technologies, USA) apparatus were applied to determine seeds oil fatty acids profile in the control, salinity‐stressed, and nanoparticles‐treated samples of the sunflower hybrids. In the GC apparatus, an HP‐5 capillary column (30 m × 0.32 mm × 0.25 μm) was employed, with helium as the carrier gas. The flow rate of the helium was maintained at a constant 1.5 mL/min. The oven temperature was initially regulated at 150°C and increased to 280°C with a heating development rate of 3°C/min, and finally kept at 280°C for 5 min. According to the user guide and manuals of Agilent company, 1 μL of each seed oil (diluted in haxan—1:20 v/v) was injected into the oven at 250°C with a split ratio of 50:1. In the GC–MS apparatus, an HP‐5MS capillary column (30 m × 0.25 mm × 0.25 μm) was used that applied helium as a carrier gas at a constant flow rate of 1.0 mL/min. The initial oven temperature was regulated at 150°C, increased to 260°C at a rate of 3°C/min, and finally kept at 260°C for 5 min. According to the user guide and manuals of Agilent company, 1 μL of each seed oil (diluted in haxan—1:20 v/v) was injected into the oven at 250°C with a split ratio of 50:1. The energy of electronic ionization was 70 eV. The obtained mass spectra were recorded for the GC and GC/MS analyses. The fatty acid profile of seed oils was determined by using the Adams library (Adams 1995) and the Wiley 7 library (NIST 17‐Wiley and Sons 2017) of mass spectra. This approach is a common method for identifying and quantifying the different fatty acids present in a sample by comparing their mass spectra to those in established databases. The relative percentages of detected compounds were calculated by normalization of the gas chromatography area (Kumar et al. 2017).

The fatty acid methyl ester types in extracted oils were determined by comparing their retention times (RT) in gas chromatography (GC) with known FAME standards, typically purchased from Sigma‐Aldrich (Ltd). This method allows for accurate identification of different FAMEs by comparing their elution times in a GC column. The product number of the used standards was arachidic acid: 39,383; behenic acid: 11,909; eicosadienoic: E3127; erucic acid: 45,629; heptadecenoic acid: H8896; lignoceric acid: L6641; linoleic acid: 62,230; γ + linolenic acid: 62,174; margaric acid: H3500; myristic acid: 70,079; myristoleic acid: 41,788; oleic acid: 75,090; palmitic acid: 76,119; palmitoleic acid: 76,169; and stearic acid: 85,679.

### 2.3 Statistical Analyses

Descriptive statistics such as average mean, minimum and maximum values, and standard deviation were calculated for each examined trait, with 20 replications. A one‐way ANOVA test was used to determine if there are any statistically significant differences (*p* ≤ 0.05) between the means of examined traits within each group. Then post hoc tests like LSD (Least Significant Difference) or Turkey's HSD (Honestly Significant Difference) were used to pinpoint which specific group means differ significantly from each other (Kholghi et al. 2011).

Box and whisker plots, also known as boxplots, were used to visually represent the distribution of a dataset, particularly highlighting the median, quartiles, and potential outliers. They effectively display how the examined characteristics change in response to different treatments or stresses by showing the spread and central tendency of data within each treatment group (Hubert and Vandervieren 2008). The Pearson correlation coefficient test was used to assess the strength and direction of linear relationships between oil fatty acid composition and seed morphological characteristics. This statistical method helps determine if changes in one variable (like fatty acid content) are associated with changes in another (like seed size), and to what extent. The coefficient, ranging from −1 to +1, indicates the nature of the relationship (positive or negative) and its strength (strong or weak) (Boddy and Smith 2009). These analyses were conducted using SPSS ver. 17.

The data was first standardized, then subjected to Principal Component Analysis (PCA) and PCA‐biplot analyses using the PAST software package to facilitate clustering. PCA is a statistical method used to reduce dimensionality in datasets, transforming correlated traits into a smaller set of uncorrelated principal components, but PCA‐biplot graphically presents the relationships between samples and characteristics in the reduced principal component space (Gewers et al. 2021).

## 3 Results

### 3.1 Morphological Investigations

The examined morphological characteristics differed among the samples. Details of the examined morphological traits, including maximum, minimum, and average values, and standard deviation were exhibited in Table 2, which provided a comprehensive overview of the examined morphological traits across the treated samples.

**TABLE 2:** The evaluated minimum, maximum, and average values, and standard deviation of the morphological characteristics in the treated/stressed samples of both hybrids on a weekly basis (all values are in mm).

Hybrid	Treatment		First week				Second week		Third week		Fourth week	
			Radicle length	Radicle diameter	Leaf length	Leaf width	Leaf length	Leaf width	Leaf length	Leaf width	Leaf length	Leaf width
G1‐601	Control	Mean	9.79 ± 5.46	1.33 ± 0.16	27.98 ± 11.79	13.85 ± 2.99	42.62 ± 16.47	20.07 ± 5.88	60.27 ± 5.00	24.97 ± 4.12	61.37 ± 4.39	18.07 ± 6.69
		Maximum	18.19	1.53	39.12	16.81	57.14	26.09	66.48	29.54	66.50	34.81
		Minimum	3.81	1.12	17.65	12.15	41.9	16.35	53.87	19.18	54.81	19.39
	Fe_2_O_3_	Mean	41.29 ± 16.54	1.81 ± 0.14	31.71 ± 3.88	15.52 ± 0.04	29.98 ± 5.86	16.78 ± 7.87	41.62 ± 23.80	21.32 ± 12.68	56.87 ± 6.43	32.58 ± 3.56
		Maximum	46.08	1.99	33.99	18.44	35.48	25.58	56.22	38.01	62.44	35.85
		Minimum	39.11	1.09	30.19	12.69	23.80	10.37	47.69	19.14	49.83	28.78
	ZnO	Mean	10.02 ± 2.65	1.46 ± 0.23	40.47 ± 4.62	14.91 ± 1.14	44.99 ± 5.01	18.43 ± 2.11	46.46 ± 13.38	23.97 ± 3.39	57.30 ± 6.27	28.00 ± 1.02
		Maximum	12.85	1.53	45.42	16.26	51.45	21.49	66.12	28.14	67.16	29.16
		Minimum	8.86	1.18	38.03	13.57	39.97	16.90	36.04	20.10	52.31	26.76
	NaCl 150	Mean	6.44 ± 1.78	1.39 ± 0.19	32.33 ± 14.30	10.78 ± 5.84	50.40 ± 12.42	19.91 ± 3.62	63.22 ± 2.68	23.17 ± 2.83	67.46 ± 5.74	27.70 ± 7.36
		Maximum	7.73	1.61	41.03	15.89	58.52	23.07	65.08	26.20	72.45	38.1
		Minimum	4.41	1.23	8.86	5.17	31.91	15.51	59.27	19.84	60.26	20.9
	NaCl 50	Mean	8.52 ± 3.50	1.68 ± 0.08	39.93 ± 1.95	15.92 ± 0.90	58.75 ± 30.30	24.24 ± 10.97	67.22 ± 23.86	27.84 ± 11.73	74.01 ± 13.35	44.01 ± 21.94
		Maximum	11.40	1.74	40.78	16.56	78.61	32.51	85.39	37.29	85.37	61.65
Shams	Control	Mean	41.50 ± 12.82	1.44 ± 0.26	37.69 ± 3.92	18.81 ± 3.52	49.34 ± 6.49	21.83 ± 2.38	51.56 ± 6.66	23.70 ± 1.65	54.82 ± 7.83	25.02 ± 2.20
		Maximum	55.00	1.78	42.88	22.60	61.00	23.94	63.12	25.79	68.42	27.95
		Minimum	17.88	1.12	32.58	14.00	43.35	17.61	45.32	22.00	46.80	22.95
	Fe_2_O_3_	Mean	37.82 ± 10.50	1.78 ± 0.36	32.18 ± 4.49	14.94 ± 2.87	44.43 ± 3.83	18.07 ± 2.16	40.03 ± 18.83	17.17 ± 6.17	48.93 ± 15.45	19.14 ± 3.45
		Maximum	48.04	1.86	39.54	18.23	48.55	20.31	54.64	23.31	61.07	23.93
		Minimum	27.04	1.27	30.42	11.46	39.46	18.01	42.50	8.67	26.53	15.35
	ZnO	Mean	29.85 ± 11.32	1.33 ± 0.29	30.89 ± 8.19	16.49 ± 4.51	43.68 ± 17.86	20.24 ± 4.10	57.37 ± 9.90	28.36 ± 4.93	60.07 ± 8.19	32.02 ± 3.01
		Maximum	47.58	1.75	40.7	23.29	65.63	23.73	65.82	33.57	69.15	34.97
		Minimum	17.44	0.95	29.48	13.92	34.45	20.04	47.81	22.7	51.7	28.4
	NaCl 150	Mean	19.36 ± 9.49	1.16 ± 0.21	28.97 ± 9.47	17.65 ± 4.87	49.38 ± 9.18	20.96 ± 2.02	54.18 ± 8.34	24.23 ± 1.78	56.68 ± 7.83	26.34 ± 2.47
		Maximum	30.13	1.46	35.71	21.61	62.44	23.13	65.91	26.59	68.57	29.25
		Minimum	5.11	0.86	10.27	9.01	41.97	17.50	46.35	22,52	50.64	23.12
	NaCl 50	Mean	21.47 ± 13.77	1.27 ± 0.24	33.66 ± 4.14	18.80 ± 3.94	45.39 ± 4.71	20.65 ± 4.41	50.33 ± 4.19	23.65 ± 5.09	51.34 ± 4.29	25.63 ± 4.50
		Maximum	39.53	1.57	39.35	24.28	52.13	28.86	54.97	33.79	55.96	34.5
		Minimum	6.21	0.95	29.91	12.6	38.83	16.24	43.59	20.05	44.25	22.04
(Continues)												

			Fifth week		Sixth week		Seventh week					
			Leaf length leaf width		Leaf length leaf width		Leaf length	Leaf width	Stem length	Stem diameter	Seed length	Seed width
G1‐601	Control	Mean	62.74 ± 7.10	31.18 ± 6.56	63.07 ± 7.21	31.29 ± 5.42	60.94 ± 5.03	32.80 ± 4.90	65.40 ± 4.27	4.12 ± 0.70	15.11 ± 2.15	5.66 ± 0.56
		Maximum	71.81	36.86	70.53	37.31	71.91	34.81	71.00	5.04	17.19	8.21
		Minimum	54.42	23.37	54.57	24.75	52.41	24.45	62.06	3.21	13.98	4.81
	Fe_2_O_3_	Mean	63.24 ± 5.15	33.61 ± 4.19	58.05 ± 4.88	29.92 ± 3.34	61.91 ± 6.31	27.08 ± 4.67	75.66 ± 15.88	4.03 ± 0.59	13.66 ± 1.21	5.71 ± 0.64
		Maximum	68.52	37.48	62.65	33.15	66.16	38.35	94.22	4.67	15.98	7.99
		Minimum	58.22	29.16	52.93	26.48	56.11	29.04	66.35	3.49	11.81	4.78
	ZnO	Mean	63.65 ± 7.29	30.55 ± 2.49	62.15 ± 8.71	30.80 ± 3.13	60.79 ± 6.67	27.05 ± 5.51	79.25 ± 5.90	3.50 ± 0.36	10.47 ± 1.09	3.71 ± 0.20
		Maximum	74.11	33.96	72.51	35.02	70.26	32.74	85.23	3.96	13.09	5.38
		Minimum	57.12	28.14	51.19	27.69	55.03	21.47	71.29	3.18	9.76	2.98
	NaCl 150	Mean	67.78 ± 7.30	29.71 ± 8.06	67.32 ± 6.18	29.97 ± 7.64	72.42 ± 9.04	36.79 ± 11.03	80.25 ± 11.02	3.57 ± 0.29	14.17 ± 2.08	5.16 ± 0.63
		Maximum	76.51	38.72	73.93	37.35	70.71	34.42	92.36	3.93	15.71	8.36
		Minimum	58.65	21.18	59.86	21.47	56.21	21.76	67.21	2.87	13.94	2.11
	NaCl 50	Mean	76.14 ± 9.26	33.23 ± 17.43	76.70 ± 10.84	45.36 ± 22.86	62.05 ± 7.58	33.37 ± 5.62	92.00 ± 25.51	4.63 ± 1.29	14.71 ± 2.19	6.53 ± 0.98
		Maximum	83.28	54.21	87.55	66.17	82.85	43.98	111.94	7.49	15.92	10.54
		Minimum	65.67	23.13	65.87	20.89	66.63	24.08	80.69	3.51	12.19	2.79
Shams	Control	Mean	46.13 ± 3.74	22.18 ± 3.30	43.24 ± 3.60	22.52 ± 3.05	42.93 ± 4.63	23.83 ± 3.51	48.83 ± 4.11	4.45 ± 0.20	8.04 ± 0.98	3.99 ± 0.21
		Maximum	50.64	27.72	47.15	27.27	50.22	27.16	54.01	4.73	10.59	5.89
		Minimum	41.08	17.81	37.28	18.29	36.47	17.79	45.00	4.24	6.60	2.78
	Fe_2_O_3_	Mean	46.88 ± 5.12	22.53 ± 4.47	47.10 ± 7.84	21.81 ± 6.88	47.33 ± 5.20	22.10 ± 3.64	51.12 ± 4.32	4.01 ± 0.21	7.87 ± 1.00	3.34 ± 0.14
		Maximum	49.06	26.32	51.85	28.13	50.94	26.93	56.01	4.31	9.96	6.89
		Minimum	38.46	16.11	35.37	12.33	39.65	18.11	45.05	3.81	5.70	2.60
	ZnO	Mean	61.08 ± 8.14	30.69 ± 4.06	49.23 ± 14.12	27.17 ± 10.19	54.26 ± 2.42	28.93 ± 4.67	62.40 ± 16.51	4.10 ± 0.85	7.88 ± 0.87	3.65 ± 0.20
		Maximum	70.41	36.22	59.75	38.21	57.03	33.49	78.05	5.21	10.12	4.71
		Minimum	53.82	26.36	24.73	11.44	51.22	24.15	43.33	3.16	6.19	3.08
	NaCl 150	Mean	56.56 ± 7.77	27.91 ± 2.85	52.27 ± 3.57	27.31 ± 2.38	52.02 ± 4.81	27.68 ± 4.14	74.16 ± 9.78	3.98 ± 0.66	6.96 ± 0.66	3.25 ± 0.25
		Maximum	70.88	31.67	57.01	30.79	56.99	31.86	84.20	4.97	7.05	6.37
		Minimum	49.61	24.15	48.51	24.22	45.79	21.56	57.01	3.26	5.69	2.15
	NaCl 50	Mean	52.02 ± 4.35	22.99 ± 3.48	46.66 ± 3.12	24.74 ± 6.33	43.04 ± 2.40	26.01 ± 1.73	54.16 ± 3.92	4.16 ± 0.57	7.45 ± 1.03	3.61 ± 0.53
		Maximum	58.06	28.55	50.43	35.71	46.41	23.77	59.11	40.98	10.98	4.76
		Minimum	45.44	17.79	41.28	18.81	40.54	19.34	49.10	3.33	6.11	3.19

#### 3.1.1 G1601 Hybrid

In the first week after treatment, the statistical analyses (ANOVA, LSD, and Tukey tests) did not detect any significant difference for root length and diameter. However, rootlet size was affected by both Fe_2_O_3_ nanoparticles and salinity. The largest (41.29 ± 5.64 mm) and widest (1.81 ± 0.14 mm) rootlets were observed in the Fe_2_O_3_ nanoparticle‐treated plants, while the smallest rootlets (6.44 ± 1.78 and 1.39 ± 0.19 mm, respectively) were found in the 150 mM salinity‐stressed plants. Conversely, leaf size was influenced differently, with the largest leaves (39.93 ± 1.95 × 15.92 ± 0.9 mm) recorded in the 50 mM salinity‐stressed plants and the smallest (27.98 ± 11.79 × 13.85 ± 2.99 mm) in the control group.

In the second (58.75 ± 30.30 × 24.24 ± 10.97 mm), third (67.22 ± 23.86 × 27.84 ± 11.73 mm), and sixth (76.70 ± 10.84 × 45.36 ± 22.86 mm) weeks, the biggest leaves belonged to the 50 mM salinity‐stressed samples, while the smallest ones (29.98 ± 5.86 × 16.78 ± 7.87 mm, 41.62 ± 23.80 × 21.32 ± 12.68 mm, and 58.05 ± 4.88 × 29.92 ± 3.34 mm, respectively) were detected in Fe_2_O_3_ nanoparticles‐treated samples.

Meanwhile, in the fourth (57.30 ± 6.27 × 28.00 ± 1.02 mm) and fifth (63.65 ± 7.29 × 30.55 ± 2.49 mm) weeks, the zinc oxide nanoparticles‐treated plants had the smallest leaves, but the biggest leaves were assigned to 50 mM salinity‐stressed plants (74.01 ± 13.35 × 44.01 ± 21.94 mm and 76.14 ± 9.25 × 33.23 ± 14.43 mm, respectively).

According to the ANOVA test, a significant difference (*p* ≤ 0.05) was detected for stem length. In this regard, the largest (92.00 ± 25.51 cm) and broadest (4.63 ± 1.29 cm) stems belonged to 150 mM salinity‐stressed plants, and the shortest (65.40 ± 4.27 cm) and thinnest (3.50 ± 0.36 cm) stems belonged to the control and ZnO nanoparticles‐treated samples, respectively.

Largest seed dimensions were detected in the control plants (15.11 ± 1.12 × 5.66 ± 0.87 mm), while the smallest (10.47 ± 0.98 × 3.71 ± 0.65 mm) ones in the zinc oxide nanoparticles‐treated plants. The ANOVA test results indicate that while most traits showed no significant difference across groups, stem length in the seventh week and leaf length in the fourth week did exhibit statistically significant differences (*p* ≤ 0.05). However, the LSD test only confirmed the significant difference in stem length of seventh week (*p* ≤ 0.05), and the Tukey test did not find any significant differences across any of the traits (Table 3). Moreover, a significant positive correlation (*r* = 0.89, *p* ≤ 0.01) was detected between seed length and seed width.

**TABLE 3:** Results of ANOVA, LSD, and Tukey analyses for the evaluated morphological traits of the hybrids.

Characteristics		Shams hybrid							G1‐601 hybrid						
		Sum of squares	df	Mean square	*F*	Sig.	LSD	Tukey	Sum of squares	df	Mean square	*F*	Sig.	LSD	Tukey
Rootlet length	Between Groups	2372.467	4	593.117	4.332	0.010	0.142	0.075	181.307	4	45.327	0.474	0.754	0.561	0.739
	Within Groups	3012.200	22	136.918					1339.321	14	95.666				
	Total	5384.667	26						1520.627	18					
Rootlet width	Between Groups	0.441	4	0.110	0.817	0.528	0.200	0.590	1.137	4	0.284	0.703	0.603	0.479	0.554
	Within Groups	2.967	22	0.135					5.663	14	0.404				
	Total	3.407	26						6.800	18					
Leaf length first week	Between Groups	235.800	4	58.950	1.796	0.166	0.292	0.127	1729.193	4	432.298	2.224	0.119	0.528	0.059
	Within Groups	722.200	22	32.827					2721.013	14	194.358				
	Total	958.000	26						4450.206	18					
Leaf width first week	Between Groups	65.450	4	16.362	1.049	0.405	0.092	0.322	220.423	4	55.106	1.855	0.174	0.275	0.167
	Within Groups	343.217	22	15.601					415.827	14	29.702				
	Total	408.667	26						636.249	18					
Leaf length second week	Between Groups	806.680	4	201.670	1.355	0.281	0.066	0.240	1356.804	4	339.201	1.810	0.183	0.096	0.83
	Within Groups	3273.617	22	148.801					2623.617	14	187.401				
	Total	4080.296	26						3980.421	18					
Leaf width second week	Between Groups	201.217	4	50.304	2.832	0.049	0.628	0.982	111.243	4	27.811	0.823	0.532	0.139	0.461
	Within Groups	390.783	22	17.763					473.283	14	33.806				
	Total	592.000	26						584.526	18					
Leaf length third week	Between Groups	752.367	4	188.092	1.970	0.134	0.302	0.061	992.787	4	248.197	1.866	0.172	0.586	0.156
	Within Groups	2100.300	22	95.468					1861.950	14	132.996				
	Total	2852.667	26						2854.737	18					
Leaf width third week	Between Groups	275.201	4	68.800	4.158	0.012	0.092	0.333	46.454	4	11.613	0.255	0.902	0.649	0.865
	Within Groups	364.020	22	16.546					638.283	14	45.592				
	Total	639.221	26						684.737	18					
Leaf length forth week	Between Groups	366.652	4	91.663	1.191	0.342	0.077	0.275	712.338	4	178.084	3.254	0.044	0.711	0.052
	Within Groups	1693.200	22	76.964					766.083	14	54.720				
	Total	2059.852	26						1478.421	18					
Leaf width forth week	Between Groups	377.763	4	94.441	9.685	0.000	1.00	0.070	633.765	4	158.441	1.659	0.215	0.237	0.215
	Within Groups	214.533	22	9.752					1336.867	14	95.490				
	Total	592.296	26						1970.632	18					
Leaf length fifth week	Between Groups	852.846	4	213.212	5.848	0.002	0.230	0.129	420.472	4	105.118	1.942	0.159	0.320	0.152
	Within Groups	802.117	22	36.460					757.633	14	54.117				
	Total	1654.963	26						1178.105	18					
Leaf width fifth week	Between Groups	317.124	4	79.281	6.223	0.002	0.201	0.683	418.015	4	104.504	1.445	0.271	0.221	0.240
	Within Groups	280.283	22	12.740					1012.617	14	72.330				
	Total	597.407	26						1430.632	18					
Leaf length sixth week	Between Groups	252.800	4	63.200	1.195	0.341	0.089	0.312	619.265	4	154.816	2.601	0.081	0.196	0.147
	Within Groups	1163.867	22	52.903					833.367	14	59.526				
	Total	1416.667	26						1452.632	18					
Leaf width sixth week	Between Groups	128.219	4	32.055	0.853	0.507	0.218	0.627	559.270	4	139.818	1.352	0.300	0.110	0.289
	Within Groups	826.300	22	37.559					1447.467	14	103.390				
	Total	954.519	26						2006.737	18					
Leaf length seventh week	Between Groups	1054.383	4	263.596	1.505	0.235	0.315	0.170	287.125	4	71.781	1.478	0.262	0.304	0.230
	Within Groups	3852.283	22	175.104					680.033	14	48.574				
	Total	4906.667	26						967.158	18					
Leaf width seventh week	Between Groups	281.367	4	70.342	1.264	0.314	0.067	0.243	287.801	4	71.950	1.812	0.183	0.410	0.248
	Within Groups	1224.633	22	55.665					555.883	14	39.706				
	Total	1506.000	26						843.684	18					
Stem length seventh week	Between Groups	2447.041	4	611.760	7.503	0.001	1.00	0.250	6103.160	4	1525.790	7.403	0.002	0.48	0.638
	Within Groups	1793.700	22	81.532					2885.367	14	206.098				
	Total	4240.741	26						8988.526	18					
Stem width seventh week	Between Groups	0.996	4	0.249	0.826	0.523	0.198	0.587	4.604	4	1.151	2.627	0.079	0.249	0.100
	Within Groups	6.633	22	0.302					6.133	14	0.438				
	Total	7.630	26						10.737	18					

**FIGURE 1:**
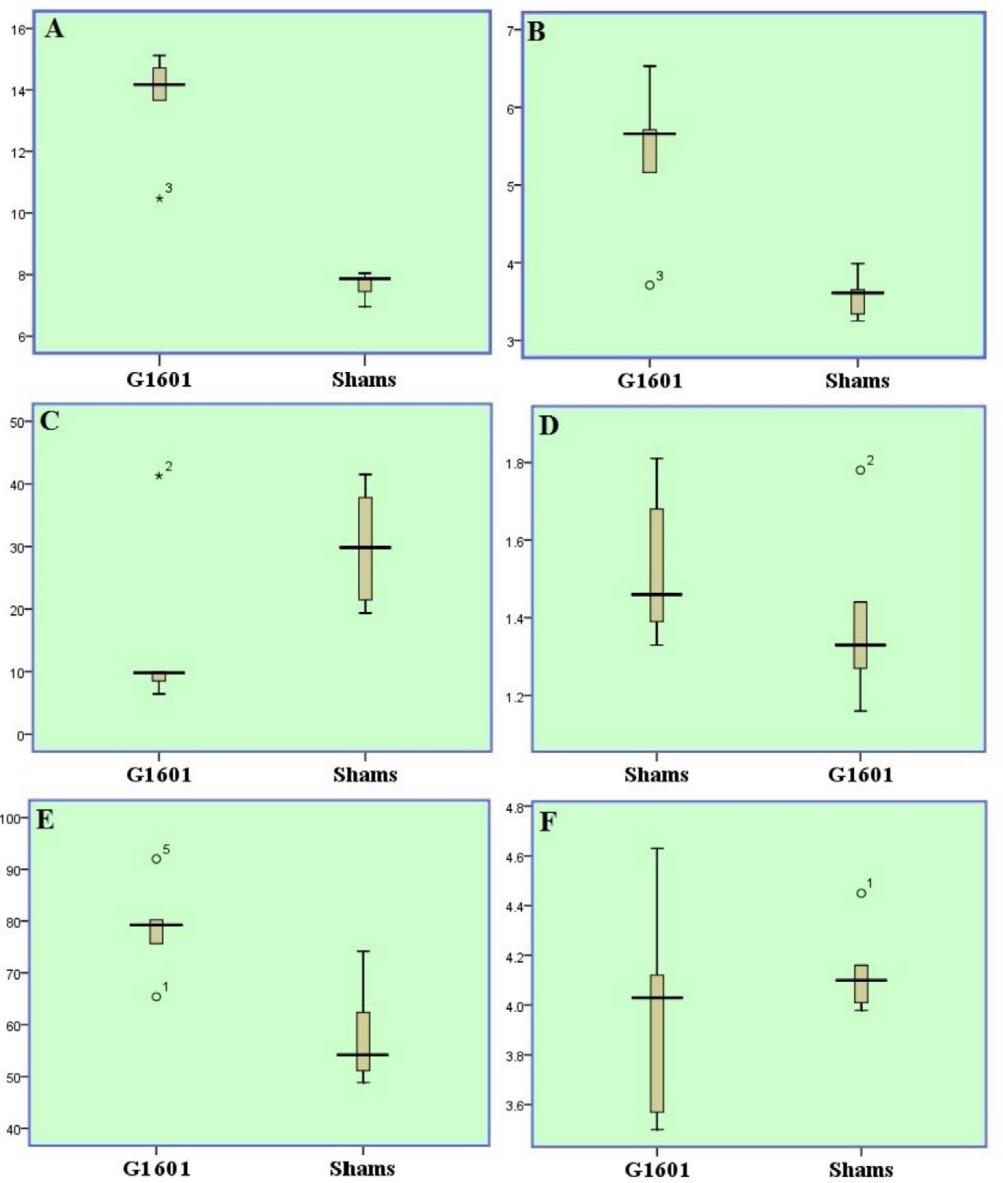
Box and whiskers plots of some main morphological characteristics. Some traits exhibited a down‐skewed (negatively skewed or left‐skewed), which means that more values clustered towards the lower end of the range, while others revealed up‐skewed (positively skewed or right‐skewed), that means that more values clustered towards the higher end of the range. (A) Seed length, (B) seed width, (C) radicle length, (D) radicle diameter, (E) stem length, and (F) stem diameter.

The box and whisker plot analysis of morphological traits for the G1601 hybrid indicated a down‐skewed (negatively skewed or left‐skewed) distribution for most characteristics, with a few exceptions, like seed length and radicle diameter exhibiting up‐skewed (positively‐ skewed or right‐skewed) distributions. This means that for most traits, the majority of the data points were clustered towards the lower end of the range, with a few larger values stretching the distribution the up. Conversely, traits like seed length and radicle diameter showed a tendency to be clustered towards the upper end of the range, with a few smaller values creating an upward tail in the distribution (Figure 1).

#### 3.1.2 Shams Hybrid

In the first week after treatment, while an ANOVA test indicated a significant difference (*p* ≤ 0.05) in rootlet length, the subsequent LSD and Tukey tests did not confirm this variation. However, the largest rootlet dimensions (41.50 ± 12.82 × 1.44 ± 0.26 mm) were observed in the control samples, while the smallest dimensions (19.36 ± 9.49 × 1.16 ± 0.21 mm) were found in the seedlings treated with 150 mM salinity stress.

Only the ANOVA test specifically identified a statistically significant difference (*p* ≤ 0.05) in leaf length during the fifth week, suggesting a notable change compared to the other weeks.

The biggest leaf dimensions in the first (37.69 ± 3.92 × 18.81 ± 3.52) and second (49.3 ± 6.49 × 21.83 ± 2.38 mm) weeks were detected in the control plants, but the smallest leaves (28.97 ± 9.47 × 17.65 ± 4.87 mm) assigned to 150 mM salinity‐stressed plants and Fe_2_O_3_ nanoparticles‐treated samples (44.34 ± 3.83 × 20.24 ± 4.10 mm), respectively. In the third (5737 ± 9.90 × 38.36 ± 4.93 mm) and fourth (60.07 ± 8.19 × 32.02 ± 3.01 mm) weeks, the zinc oxide nanoparticles‐treated samples had the biggest leaves, while their smallest dimensions belonged to the Fe_2_O_3_ nanoparticles‐treated plants (40.03 ± 18.83 × 17.17 ± 6.17 and 48.93 ± 15.45 × 19.14 ± 3.45 mm, respectively). In the fifth week, the biggest (61.08 ± 8.14 × 30.69 ± 4.06 mm) and smallest (46.13 ± 3.74 × 22.18 ± 3.30 mm) leaf dimensions were detected in the zinc oxide nanoparticles‐treated and the control plants, respectively. The same conditions hold true for plants in the seventh week, and zinc oxide nanoparticles‐treated samples had the biggest leaf dimensions (54.26 ± 2.42 × 28.93 ± 4.67 mm), while the smallest (42.93 ± 4.63 × 23.83 ± 3.51 mm) ones belonged to the control plants.

The ANOVA test indicated a significant difference (*p* ≤ 0.05) in stem length, but LSD and Tukey analyses did not. Additionally, plants subjected to 150 mM salinity stress exhibited the largest stem dimensions (74.16 ± 9.78 × 3.98 ± 0.66 cm), while control plants displayed the smallest (48.83 ± 4.11 × 4.45 ± 0.20 cm). The control plants typically exhibited the largest seed dimensions (8.04 ± 0.98 × 3.99 ± 0.21 mm), while plants under 150 mM NaCl stress often have smaller (6.96 ± 0.66 × 3.25 ± 0.25 mm) seeds.

The ANOVA test revealed significant variation (*p* ≤ 0.05) for a subset of morphological traits (rootlet length and leaf width at Weeks 2–5, stem length at Week 7, and leaf length at Week 5), despite not showing significant differences for most examined traits. However, post hoc tests like LSD and Tukey did not confirm these significant differences, suggesting the ANOVA's significant findings might be due to other factors not captured by the post hoc tests (Table 3).

**FIGURE 2:**
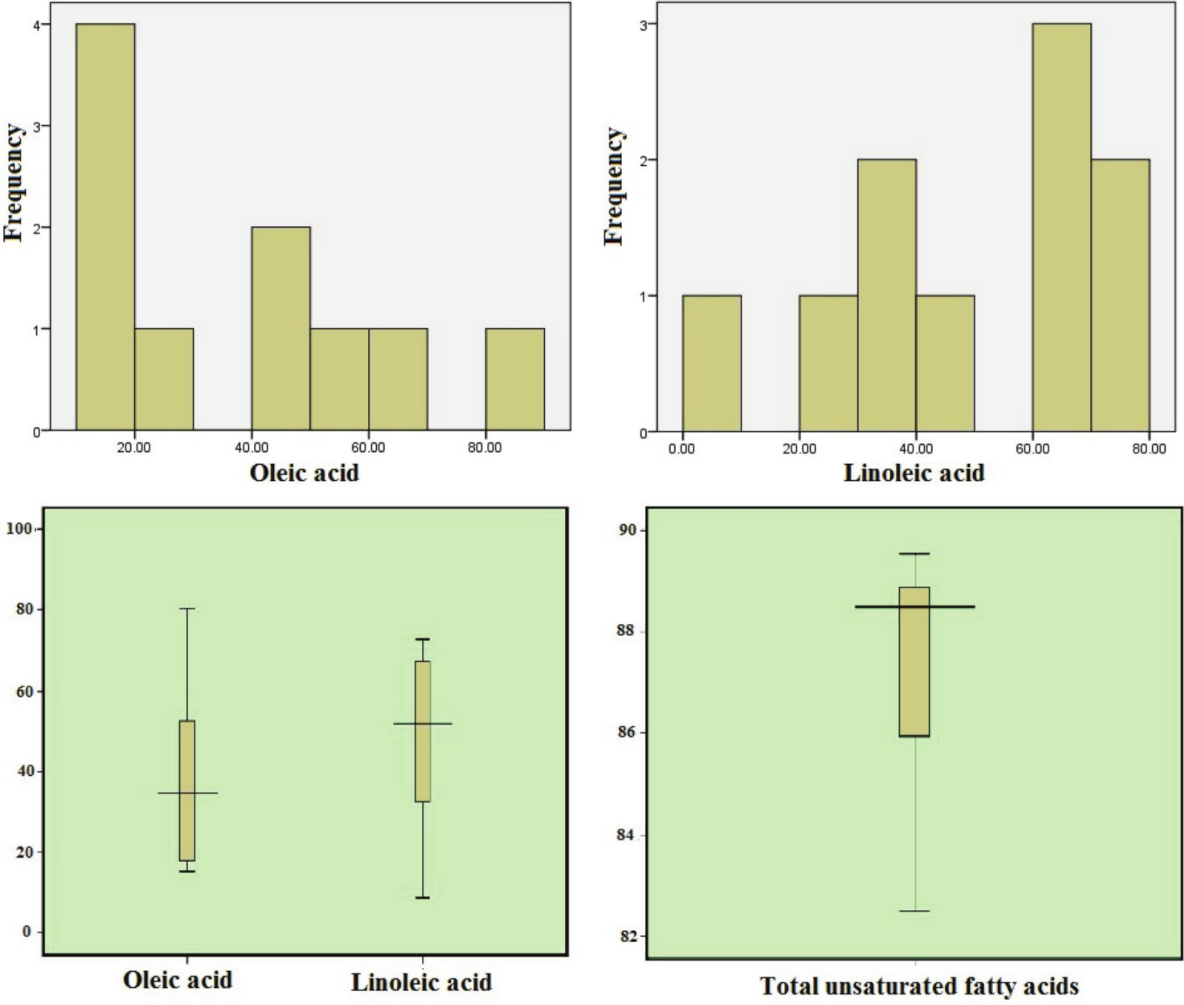
Box and whiskers plots explored the main fatty acids of the evaluated hybrids. Linoleic acid and total unsaturated fatty acid amounts showed down‐skewed distribution (negatively skewed or left‐skewed) which means the majority of values are towards the lower end of measurement, while oleic acid exhibited up‐skewed (positively skewed or right‐skewed) distribution.

A strong positive correlation (*r* = 0.83, *p* ≤ 0.05) existed between seed length and seed width, indicating that as one increased, the other tended to increase as well. Furthermore, the data in the box and whisker plot revealed specific skewness patterns. Seed length and width were negatively skewed (down skewed or left‐skewed), meaning the tail of this distribution was towards the lower end of the range. Conversely, radicle diameter and stem length were positively skewed (up skewed or right‐skewed), with the tails towards the upper end of the range. The remaining characteristics exhibit a symmetrical distribution (Figures 1 and 2).

### 3.2 Seed Oil Fatty Acids Profile

The fatty acid profiles of different hybrids and their treated samples were summarized in Table 4, which provided a comparison of the percentage of each fatty acid in the samples.

**TABLE 4:** Seed oils fatty acid profiles of the nanoparticles and salinity stressed sunflower hybrids.

Fatty acid name	Formula	Shams					G1601				
		Control	50 mM	150 mM	Zn	Fe	Control	50 mM	150 mM	Zn	Fe
Myristic acid	C14:0	0.06	0.09	0.11	0.30	0.07	0.02	0.03	0.02	0.20	0.03
palmitic acid	C16:0	7.28	7.32	8.97	9.75	7.32	3.75	4.96	4.22	8.87	5.45
Margaric acid	C17:0	0.05	0.05	0.03	0.05	0.04	0.04	0.05	0.06	‐‐‐‐‐‐	0.06
Stearic acid	C18:0	3.30	2.76	3.63	5.78	2.48	4.60	4.14	4.84	6.00	5.42
Arachidic acid	C20:0	0.20	0.25	0.35	0.56	0.26	0.40	0.32	0.38	0.70	0.36
Behenic acid	C22:0	0.51	0.54	0.59	0.80	0.55	1.28	1.08	1.27	1.21	1.05
Lignoceric acid	C24:0	0.25	0.28	0.39	0.37	0.36	0.34	0.31	0.34	0.47	0.28
Total of saturated		11.65	11.29	14.07	17.61	11.08	10.43	10.89	11.13	17.45	12.65
Myristoleic Acid	C14:1	0.02	‐‐‐‐‐‐	0.04	0.07	0.02	0.02	0.01	0.01	‐‐‐‐‐‐	0.02
Palmitoleic acid	C16:1	0.17	0.12	0.39	0.27	0.30	0.09	0.07	0.07	0.17	0.09
*trans‐10‐*Heptadecenoic acid	C17:1	0.05	0.03	0.04	0.03	0.06	0.04	0.04	0.03	‐‐‐‐	0.04
Oleic acid	C18:1	17.71	15.33	17.93	18.43	22.78	80.34	52.49	64.34	49.47	46.54
Linoleic acid	C18:2	70.11	72.98	67.19	63.29	65.44	8.67	36.11	24.10	32.44	40.29
γ‐Linolenic acid	C18:3	0.06	0.06	0.07	0.16	0.07	0.09	0.12	0.10	‐‐‐‐‐‐	0.13
Gondoic acid	C20:1	0.11	0.10	0.07	0.07	0.13	0.25	0.20	0.17	0.15	0.16
eicosadienoic acid	C20:2	0.02	‐‐‐‐‐‐	0.09	0.80	0.04	0.01	‐‐‐‐‐‐	‐‐‐‐‐‐	0.29	‐‐‐‐‐‐
Erucic acid	C22:1	0.03	0.02	0.12	0.03	0.02	‐‐‐‐‐‐	0.01	‐‐‐‐‐‐	‐‐‐‐‐‐	0.01
Nervonic acid	C24:1	0.01	0.02	‐‐‐‐‐‐	‐‐‐‐‐‐	0.01	0.02	0.01	‐‐‐‐‐‐	‐‐‐‐‐‐	0.01
Total of unsaturated		88.29	88.66	85.94	83.15	88.87	89.53	89.06	88.82	82.52	87.29
Other		0.06	0.05	‐‐‐‐‐‐	0.04	0.05	0.04	0.05	0.05	0.03	0.06

*Note:* The information presented included the names, chemical formulas, and percentages of both saturated and unsaturated fatty acids found in samples. Dotted lines indicates the absence of specific fatty acids in the examined samples.

#### 3.2.1 Shams Hybrid

In the Shams hybrid, the seed oil composition included both saturated and unsaturated fatty acids. Regardless of treatments (control or stress/treament), a majority of the oil consisted of unsaturated fatty acids, ranging from 83.15% to 88.87%. Specifically, ZnO nanoparticle‐treated plants had 83.15% unsaturated fatty acids, while Fe_2_O_3_ nanoparticle‐treated plants had 88.87% unsaturated fatty acids.

Linoleic acid, a major unsaturated fatty acid, had the highest concentration (72.98%) in 50 mM salinity‐stressed plants, and the lowest (63.29%) in those treated with ZnO nanoparticles. Oleic acid, the second most abundant unsaturated fatty acid, varied from 15.33% (in ZnO nanoparticle‐treated plants) to 22.78% (in Fe_2_O_3_ nanoparticle‐treated plants).

The analysis of fatty acids in various oil samples revealed the presence of several unsaturated fatty acids in trace amounts, with most being common to all samples. However, myristic acid, nervonic acid, and eicosadienoic acid were not found in all samples, indicating variations in the composition of the treated/stressed oils.

The study revealed that total saturated fatty acids varied in a range of 11.08% (Fe_2_O_3_ nanoparticles) to 17.61% (ZnO nanoparticles). Palmitic acid was the dominant saturated fatty acid, with the highest concentration (9.75%) in ZnO nanoparticles‐treated plants and the lowest (7.28%) in the control samples. Stearic acid was detected as the second main saturated fatty acid in a range of 2.48% to 5.78% that were registered in Fe_2_O_3_ and ZnO nanoparticles‐treated plants, respectively. A negative significant correlation was detected (*r* = −0.93, *p* ≤ 0.05) between oleic acid and linoleic acid percentages.

#### 3.2.2 The G1601 Hybrid

The seed oils primarily consisted of unsaturated fatty acids, with oleic and linoleic acids being the most abundant. The amounts of these fatty acids differed significantly between various samples, with the control sample showing the highest unsaturated fatty acid amount (89.53%) and the ZnO nanoparticle treated plants having the lowest (82.52%).

The percentage of oleic acid, as the primary component, varied in a range of 46.54% (Fe_2_O_3_ nanoparticles‐treated samples) and 80.34% (the control samples). Linoleic acid amounts, as the second one, varied among the samples and were detected in a range of 8.67% (the control samples) to 40.29% (Fe_2_O_3_ nanoparticles‐treated samples). Additionally, several unsaturated fatty acids were identified in a trace amount. However, most of them were common, appearing in all samples (such as palmitoleic and gondoic acids) or most samples (e.g., myristoleic and γ‐linolenic acids), and a few were found less frequently (for instance, erucic, and eicosadienoic acids).

In seed oils, saturated fatty acids, primarily palmitic and stearic acids, varied in concentration between the control samples and those treated with ZnO nanoparticles. The control samples had 10.43% saturated fatty acids, while treated plants showed 17.45%. Palmitic acid ranged from 3.75% (the controls) to 8.87% (ZnO nanoparticle‐treated plants), and stearic acid ranged from 4.14% in salinity‐stressed samples to 6.00% in ZnO nanoparticle‐treated plants. Margaric acid was notably absent in the ZnO nanoparticle‐treated plants.

Salinity stress negatively correlates with oleic acid content (*r* = −0.87, *p* ≤ 0.05). Furthermore, there was a strong negative correlation between linoleic and oleic acid contents (*r* = −0.98, *p* ≤ 0.01).

**FIGURE 3:**
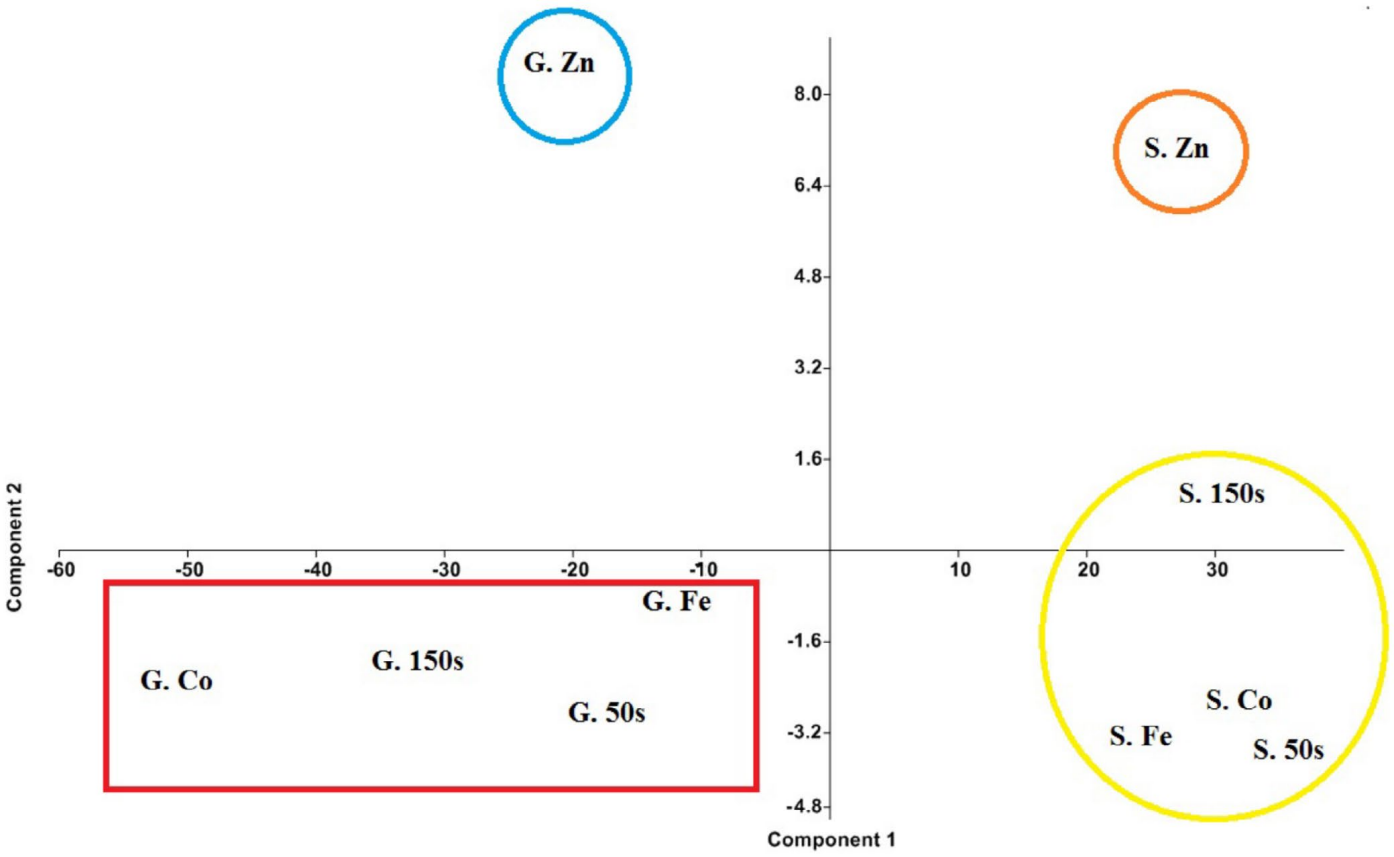
PCA plot of the salinity and nanoparticle‐treated sunflower hybrids. This plot effectively visualizes the impact of salinity stresses, zinc oxide and iron oxide nanoparticle treatments on different sunflower hybrids. Component 1, explaining 98% of the variation, separated samples based on hybrid type, which indicated that the sunflower hybrid is the primary factor differentiating the samples. Then, component 2, with 1.8% of the variance, further distinguished each hybrid into zinc oxide nanoparticle‐treated and remaining groups, which highlighted the significant influence of zinc oxide nanoparticle treatment on phytochemical traits. Abbreviations: G.Co: G1601 control, G.50s: G1601 50 mM salinity‐stressed, G.150s: G1601 150 mM salinity‐stressed, G.Fe: G1601 Iron oxide nanoparticle‐treated, G.Zn: G1601 Zinc oxide nanoparticle‐treated, S.Co: G1601 control, S.50s: G1601 50 mM salinity‐stressed, S.150 s: G1601 150 mM salinity‐stressed, S.Fe: G1601 Iron oxide nanoparticle‐treated, S. Zn: G1601 Zinc oxide nanoparticle‐treated.

PCA performed on chemical data to reduce dimensionality and identify principal components (PC). Nine components were detected, which contained 100% of the total variances. The first (98.148%) and second (1.8077%) components were used to create a scatter plot (Table 5), where PC1 separated treated/stressed samples of both hybrids, and PC2 divided samples of each hybrid into two groups. Notably, zinc oxide nanoparticle‐treated samples were distinctly separated from other samples of each hybrid (Figure 3).

**TABLE 5:** Principal component analysis of the seed oils fatty acids of the treated samples.

Principal components	Eigenvalue	% variance
1	1046.82	98.148
2	19.28	1.8077
3	0.145283	0.038936
4	0.0418062	0.0039197
5	0.00881665	0.00082663
6	0.00383286	0.00035936
7	0.000722608	0.0000677505
8	0.000416793	0.00003907805
9	0.000194651	0.00001825078

*Note:* In this analysis, nine principal components were extracted from the phytochemical traits, with the first two components, which have the highest eigenvalues and variance, being used to create a 2D plot for sample comparison.

## 4 Discussion

The regular sunflower oils containing a high percentage of linoleic acidand are more suitable to use as a salad dresser. These oils are not ideal to utilize as deep‐frying oil due to weak oxidative stability (Chernova et al. 2021). Therefore, developing new hybrids with modified oil profiles is crucial for meeting specific needs, including improved health benefits, industrial applications, and sustainable agriculture. This can be achieved through mutation, breeding, and hybrid development, or by manipulating cultivation practices to influence seed oil composition (Feng, Jan, and Seiler 2022; Feng, Kreslavski, et al. 2022).

Sunflower hybrids are primarily categorized into two main types: oilseed sunflowers and non‐oilseed sunflowers. Oilseed sunflowers are grown for their high oil content and are used for oil extraction, while non‐oilseed sunflowers, also known as confectionery sunflowers, are grown for their large seeds, which are consumed as nuts (Talebi et al. 2024). The Shams hybrid is associated with the oilseed type, and G1601 is a specific hybrid of the non‐oilseed type.

The sunfloer hybrids were further categorized into three subclasses based on their oleic acid contents: standard (< 50% oleic acid), mid‐oleic (50%–70% oleic acid), and high‐oleic (over 80% oleic acid). This classification is important because the oleic acid content significantly impacts the oil's properties and applications (Manalili et al. 2021). The G1601 hybrid, initially categorized as high oleic acid, experienced a shift in fatty acid composition when exposed to salinity stresses or nanoparticle treatments. Specifically, the oleic acid content decreased, while the linoleic acid content increased, indicating a change in the seed's fatty acid profile under these conditions.

This shift in fatty acid composition is likely due to the plant's response to environmental stresses. Salinity and nanoparticle treatments can induce various physiological changes in plants, including alterations in enzyme activity related to fatty acid biosynthesis. The increase in linoleic acid, which is a polyunsaturated fatty acid, could be a consequence of the plant's attempt to adapt to the stress conditions. This fatty acid is a common component of plant cell membranes. An increase in linoleic acid amount is often observed in plants exposed to stress, suggesting it plays a key role in the plant's stress response (He and Ding 2020). Similar results were reported by Céccoli et al. (2022), in which the salinity stress decreased oleic/linoleic acids ratio in four sunflower hybrids (ACA885, SRM779CL, TM, and SRM769). While a reverse pattern was reported by Flagella et al. (2004), whereas salinity stress enhanced the oleic acid amount and decreased linoleic acid percentage in oleic hybrids of sunflower.

Therefore, it seems that the effects of salinity stress on oleic and linoleic acids levels differ significantly between diverse hybrids of sunflower. Some hybrids may explore an increase in linoleic acid under salt stress, while others might represent a decrease or even an increase in oleic acid amount. There is no universal rule, and the specific response depends on the plant's genetic structure and plants adaptation to stress. Understanding these hybrid‐specific responses is important for breeding programs aimed at developing salt‐tolerant sunflower varieties with desirable fatty acid profiles for specific oil applications. For example, some research suggested that linoleic acid is more desirable for certain uses like margarine and dressings, while oleic acid is better for high‐temperature cooking and frying due to its stability (Cucci et al. 2007).

Oleic acid percentages in the G1601 hybrid plants decreased under salinity stress and nanoparticle treatment, with iron oxide nanoparticles causing the lowest percentage. According to Zhang et al. (2014), the delta‐12 oleate desaturase gene (*FAD2*‐1) converts oleic acid into linoleic acid. This is one of the key enzymes that determines the fatty acid profile of seed oil. It seems that the application of different levels of salinity stress, zinc oxide, and iron oxide nanoparticle treatments in the G1601 hybrid increases the expression of the *FAD2*‐1 gene and converts oleic acid into linoleic acid. Hernández et al. (2011) suggested that the *FAD2* gene plays a prominent role in regulating the composition of lipids in the intracellular membranes. Additionally, it regulates the biophysical properties and proper function of membrane‐attached proteins in the salinity‐stressed plants.

Environmental conditions significantly influence the amount of oleic acid in plants, particularly during the grain‐filling period. Factors such as temperature, solar radiation, humidity, day length, and moisture availability can all affect the oil contents and the proportion of oleic acid within those oils (Zuil et al. 2012). For example, Echarte et al. (2010) specifically highlighted that minimum night temperature and intercepted solar radiation have an additive effect on the oleic acid percentage in sunflower oil. This implies that both factors, when increased, contribute to a greater overall increase in oleic acid content. Alberio et al. (2017) reported similar results, where the oleic acid percentage in the oil of Pervenent (high oleic acid containing sunflower) hybrid varied in a range of 15%–91%, which significantly correlated with the temperature during the reproductive phase of plants. Moreover, the sowing time, humidity, and rainfall affect the percentage of oleic acid in sunflower oils (Akkaya et al. 2019).

The Shams sunflower hybrid, classified within standard sunflower types, exhibits an increased oleic acid content under various treatments, except for 50 mM salinity‐stressed samples. A study on sunflower hybrids (Alberio et al. 2016) suggested that induced mutations or breeding programs, specifically those affecting oleic acid composition, can significantly influence how each cultivar responds to different stress conditions. Furthermore, the current study indicated that this influence is not uniform; the changes in oleic acid composition due to these interventions vary depending on the type of stress encountered.

Due to time and budget limitations, we were unable to investigate the expression of genes involved in the biosynthesis of the main fatty acids of seed oils. While, according to former investigations (Rauf 2019; Dimitrijević et al. 2017), the biosynthesis of high oleic acid percentage was controlled by a single dominant mutated gene. Additionally, salinity stress can influence the expression of Δ‐12 fatty acid desaturase genes at the transcriptional level (Zhang et al. 2014).

When plants are subjected to increasing levels of salinity stress, the content of linoleic acid tends to diminish in both hybrids. This reduction in linoleic acid is often accompanied by an increase in other fatty acids, such as oleic acid. Ghorbannia‐Delavar et al. (2023) suggested that under drought stress conditions, moderate stress increased the amount of linoleic and linolenic acids, whereas the level of linolenic and linolenic acids reached the lowest level under severe drought stress. A similar result was reported for two sunflower hybrids. Di Caterina et al. (2007) found that under severe salt stress conditions, sunflower plants, specifically the hybrids Tenor (high oleic acid type) and Carlos (standard type), showed a significant increase in oleic acid content while experiencing a decrease in linoleic acid content. This suggests that salinity is likely inhibiting the enzyme oleate desaturase, which is responsible for converting oleic acid into linoleic acid in developing seeds (Sarmiento et al. 1998).

In shams hybrid, linoleic acid percentages generally decreased in samples treated with nanoparticles and salinity, except in plants stressed by 50 mM salinity. The G1601 hybrid revealed an opposite trend, with the highest linoleic acid amount observed in iron oxide nanoparticles‐treated plants. In experiments with sunflower hybrid SY Neostar, the application of iron oxide nanoparticles resulted in a decrease in oleic acid content and a corresponding increase in linoleic acid content compared to the control group. This suggests a potential shift in the fatty acid profile of sunflower oil due to nanoparticle treatment (Ernst et al. 2023).

This suggested that the response to nanoparticles and salinity treatments varies depending on the plant hybrid, the specific stress level, and nanoparticle type. Similar results were reported by Batool et al. (2021) where Ag nanoparticle treatment significantly increased linoleic acid amount in sunflower oil. According to former investigations, some environmental parameters can influence linoleic acid amount in seed oils. For example, Flagella et al. (2002) indicated that the linoleic acid percentage significantly correlated with the cultivation time and early sowing increases linoleic acid percentage in seed oil.

The induced salinity stress and nanoparticle treatments significantly impacted the total unsaturated fatty acids in both hybrids, with the lowest amounts observed in the zinc oxide nanoparticles‐treated samples. ZnO nanoparticles likely reduce unsaturated fatty acids through a combination of oxidative stress, altered enzyme activity, and other cellular interactions. These nanoparticles can induce the production of reactive oxygen species (ROS), leading to oxidative stress, which can damage cellular components, including unsaturated fatty acids. Additionally, ZnO nanoparticles can interfere with the activity of enzymes involved in fatty acid metabolism and degradation (Saliani et al. 2016).

Safavi et al. (2018) examined the effects of zinc oxide and iron oxide nanoparticles on the oil profile of *Pleurotus ostreatus* (Jacq. ex Fr.) P. Kumm. and detected that ZnO nanoparticles strongly and Fe_2_O_3_ nanoparticles slightly diminished the total unsaturated fatty acids amount. Unsaturated fatty acids offer nutritional benefits and contribute to certain desirable qualities in oils, while decreasing their overall amounts can improve oils stability and shelf life. This is because unsaturated fatty acids are more susceptible to oxidation that leads to rancidity and a decrease in quality.

In contrast to the G1601 hybrid, the Shams hybrid explored a decrease in the percentage of total polyunsaturated fatty acids when treated with nanoparticles. This indicates that these nanoparticles had an inverse effect on polyunsaturated fatty acid levels in these particular hybrids. The similar results were reported in some previous investigations (Safavi et al. 2018; Ogwok et al. 2017).

Nanoparticle treatment and salinity stress can alter the fatty acid composition of sunflower oil, impacting its quality and stability. Oils with high polyunsaturated fatty acid contents are more prone to oxidation, while those with higher saturated or mono‐unsaturated fatty acid amounts are more stable during cooking and have a longer shelf life (Romano et al. 2021). However, saturated fatty acids can also contribute to increased cholesterol levels (Astrup et al. 2021).

The PCA analysis on the seed oil fatty acid composition grouped together samples of each hybrid. The most notable finding was the distinct separation of zinc oxide nanoparticle‐treated samples from other samples of each hybrid. This suggested that these nanoparticles have a significant impact on the chemical composition, making them distinct from other members of the same hybrid. The observation that zinc oxide‐treated nanoparticle samples of each hybrid exhibited a higher total saturated fatty acid content compared to other samples suggested that zinc oxide treatment significantly alters the fatty acid profile, potentially differentiating these specific samples. This increase in saturated fatty acids could have implications for the overall quality and characteristics of the treated samples. A similar result was reported by Ma et al. (2025). ZnO nanoparticles can enhance the bioavailability of zinc, which is a cofactor for enzymes involved in fatty acid desaturation. Desaturation is the process of converting saturated fatty acids into unsaturated fatty acids. By increasing zinc availability, these nanoparticles might indirectly inhibit this conversion, potentially leading to a higher proportion of saturated fatty acids.

For detecting significant differences among morphological traits, ANOVA, LSD, and Tukey tests were performed, while only the ANOVA test revealed significant differences for a few traits. This often occurs because the ANOVA test is more sensitive to detecting overall differences, while post hoc tests are more conservative in controlling for Type I error (false positives). Moreover, the ANOVA test might detect a small but statistically significant difference that is not practically meaningful, while the post hoc tests require a larger difference to be considered significant (Gurvich and Naumova 2021).

For both hybrids, the first and second largest rootlet dimensions (length and width) belonged to the nano‐priming seeds. Gupta et al. (2024) suggested that some growth factors, including plant biomass, root development, leaf size, total phenolic and anthocyanin contents, and antioxidant activity, significantly increased in the iron oxide nanoparticles‐priming sunflowers. It seems that these nanoparticles develop photosystem I and II activities in sunflower plants. Nile et al. (2022) suggested that nano‐priming is one of the effective approaches to seed priming, which develops seed capability to uptake nutrients and renovate seed metabolism. Additionally, nanoparticle treatment positively influences seedling traits, such as vigor index, root formation, and seedling growth in diverse plant species (Abbasi‐Khalaki et al. 2021).

Results exhibited that seed priming with iron oxide nanoparticles was more effective than zinc oxide nanoparticles. Sundaria et al. (2018) also reported similar results. Iron oxide nanoparticles, when used as a treatment, can hinder leaf growth, particularly in the G1601 hybrid during the second, third, and sixth weeks, and in the Shams hybrid during the first to fourth weeks. The exact mechanisms by which iron oxide nanoparticles inhibit leaf growth are still being investigated. They could involve altered cell division, reduced chlorophyll synthesis, or interference with nutrient transport (Gowtham et al. 2024).

These results explored that nanoparticles effect is stronger on certain hybrids and during specific time periods, and indicated that the interaction between iron oxide nanoparticles and plant growth is complex and may be influenced by various factors, including plant genotype (hybrid type), age, and developmental stage.

In plants of the G1601 hybrid, a moderate level of salinity stress (50 mM) caused a notable increase in leaf size, except for those treated during the seventh week. The findings suggest that a moderate salinity stress can induce leaf enlargement in this specific hybrid. Bakhoum et al. (2020) suggested that a moderate level of salinity stress increased the dimensions of some morphological characteristics in sunflower plants, including shoot length, number of leaves, stem circumference, and shoot fresh and dry weights. Additionally, the Indole‐3‐acetic acid (IAA) content significantly increased in sunflower plants under moderate salinity stress. This hormone develops the defense system of plants against environmental stress through antagonistic or synergistic interaction with other growth regulators (Abass and Mohamed 2011). Similar results were reported for other taxa; for example, 50 mM NaCl salinity stress significantly increased growth parameters in *Crithmum maritimum* L.—Apiaceae (Vahdati et al. 2012), *Lotus creticus* L.–Fabaceae (Rejili et al. 2007), and *Centella asiatica* (L.) Urban‐Apiaceae (Hoang and Rehman 2023).

Salinity stress significantly impairs root growth and development in plants compared to non‐stressed controls. This stress creates multiple challenges which inhibit root growth and development. These include osmotic stress, ion toxicity, nutrient imbalances, and cellular changes, all of which contribute to decreased root size and function under saline environments (Chaudhary et al. 2024). Moreover, salinity stress can also lead to changes in auxin gradients within the root, potentially impacting the development of lateral roots and other root structures in contrast to the enhancing effect of IAA on cell expansion in upper plant organs (Edelmann 2022). Moreover, Ismail et al. (2024) indicated that high salinity stress can negatively impact sunflower growth by reducing leaf area and root length. This is likely due to chloride ion accumulation in the roots, which can have a toxic effect on both root and leaf development.

The biggest leaves dimensions in the third to fifth and seventh week belonged to zinc oxide nanoparticle‐treated samples of shams hybrid. This suggested a positive impact of zinc oxide nanoparticles on leaf growth in this specific hybrid. Similar results were reported by Amin‐Jafari et al. (2024), who indicated that Zn fertilization improves leaf area index, yield (grain weight), and physiology (water use efficiency and protein percentage). However, nanoparticles type and genetic structure (hybrid type) are the determining factors. The interactions between nanoparticles and plants are influenced by both the specific properties of the nanoparticles, such as their sizes, shape, surface chemistry, and the inherent genetic characteristics of the plant. These factors collectively determine the extent and nature of nanoparticle uptake, translocation, and their impacts on plant physiology and gene expression (Wang et al. 2023).

The box and whisker plots would visually show that G1601 hybrid generally had higher values for most traits than Shams hybrid, indicated by a higher median and/or larger quartiles. In addition, the longer whisker on the lower end suggested a wider range of lower values in G1601 hybrid, indicating a skew towards the lower end of the distribution. This condition could be due to outliers or simply a wider spread of values towards the lower end of the scale. The Shams hybrid may have a more balanced or different distribution of traits in box and whisker plots.

It is recommended to investigate the effects of various nanoparticles on plant growth and health, particularly under different salinity levels, by examining morphological traits and fatty acid composition. This research should explore a range of nanoparticle types, concentrations (including standard and elevated levels), and salinity conditions to understand their impact on plant response. Examining the expression of genes involved in seed oil biosynthesis is crucial for understanding and potentially improving seed oil quality and quantity. By analyzing gene expression, breeders can identify key enzymes and regulatory pathways involved in the synthesis of fatty acids.

## 5 Conclusion

The study investigated the effects of iron oxide and zinc oxide nanoparticles, alongside salinity stresses, on two sunflower hybrids (G1601 and Shams). Salinity stresses increased leaf size in G1601 samples, while nanoparticle treatments decreased it. In the Shams hybrid, Fe_2_O_3_ nanoparticle treatment initially reduced leaf size, while ZnO nanoparticles increased it in later weeks. Despite these trends, statistical analysis (ANOVA, Tukey, and LSD) showed no significant difference for most morphological traits. G1601 was classified as a high oleic acid hybrid, and linoleic acid was found in a lower concentration, while Shams was a standard type, with linoleic acid dominating its seed oil profile. Under various nanoparticle treatments and salinity stress conditions, the percentages of both saturated and unsaturated fatty acids in the examined hybrids changed. Sunflower responses varied based on genetic structure (hybrid type), age, treatment type, and concentration. To fully understand how salinity/nanoparticle treatments induce variations in morphological and phytochemical traits, further research is needed to identify the underlying genetic basis. This research should focus on analyzing the genetic diversity and gene expression patterns in these hybrids under stress conditions, utilizing techniques like transcriptomics and genome‐wide association studies.

## Author Contributions

**Shiva Shariatzadeh:** data curation (equal), investigation (equal), visualization (equal), writing – original draft (equal). **Seyed Mehdi Talebi:** conceptualization (equal), software (equal), supervision (equal), writing – original draft (equal). **Kimia Anjomani:** conceptualization (equal), software (equal), validation (equal), writing – original draft (equal). **Mansour Ghorbanpour:** software (equal), writing – review and editing (equal).

## Ethics Statement

The authors have nothing to report.

## Consent

We hereby declare that we participated in this study and the manuscript's development. The final version of the paper has been checked and we give consent for publication.

## Conflicts of Interest

The authors declare no conflicts of interest.

## Data Availability

The raw data will be available from the corresponding author on reasonable request from the corresponding authors.
